# Testis-specific transcriptional regulators selectively occupy BORIS-bound CTCF target regions in mouse male germ cells

**DOI:** 10.1038/srep41279

**Published:** 2017-02-01

**Authors:** Samuel Rivero-Hinojosa, Sungyun Kang, Victor V. Lobanenkov, Gabriel E. Zentner

**Affiliations:** 1 Molecular Pathology Section, Laboratory of Immunogenetics, National Institute of Allergy and Infectious Diseases, National Institutes of Health, Rockville, MD 20852, USA; 2 Department of Biology, Indiana University, Bloomington, IN 47405, USA

## Abstract

Despite sharing the same sequence specificity *in vitro* and *in vivo*, CCCTC-binding factor (CTCF) and its paralog *b*rother *o*f the *r*egulator of *i*mprinted *s*ites (BORIS) are simultaneously expressed in germ cells. Recently, ChIP-seq analysis revealed two classes of CTCF/BORIS-bound regions: single CTCF target sites (1xCTSes) that are bound by CTCF alone (CTCF-only) or double CTCF target sites (2xCTSes) simultaneously bound by CTCF and BORIS (CTCF&BORIS) or BORIS alone (BORIS-only) in germ cells and in BORIS-positive somatic cancer cells. BORIS-bound regions (CTCF&BORIS and BORIS-only sites) are, on average, enriched for RNA polymerase II (RNAPII) binding and histone retention in mature spermatozoa relative to CTCF-only sites, but little else is known about them. We show that subsets of CTCF&BORIS and BORIS-only sites are occupied by several testis-specific transcriptional regulators (TSTRs) and associated with highly expressed germ cell-specific genes and histone retention in mature spermatozoa. We also demonstrate a physical interaction between BORIS and one of the analyzed TSTRs, TATA-binding protein (TBP)-associated factor 7-like (TAF7L). Our data suggest that CTCF and BORIS cooperate with additional TSTRs to regulate gene expression in developing male gametes and histone retention in mature spermatozoa, potentially priming certain regions of the genome for rapid activation following fertilization.

CTCF is a ubiquitously expressed chromatin-binding factor that contains 11 zinc fingers (ZFs) and is conserved from *Drosophila* to human^1^,^2^. CTCF regulates transcription through promoter binding^3^,^4^ as well as methylation-sensitive insulator activity of the H19 imprinting control region, where it blocks enhancer looping to potential target promoters^2^,^5^,^6^,^7^ and can directly interact with the largest subunit of RNA polymerase II (RNAPII) to tether it to certain CTCF target sites (CTSes) in promoters and enhancers^8^. CTCF has also been implicated in numerous other chromatin-based processes, including RNAPII pausing^9^, regulation of ribosomal RNA transcription^10^,^11^,^12^, X chromosome inactivation^13^,^14^, and the formation of higher-order chromatin structures^15^,^16^,^17^. In addition to CTCF, mammals also express the paralogous protein *b*rother *o*f the *r*egulator of *i*mprinted *s*ites (BORIS, also known as CTCFL)^18^. While CTCF is ubiquitously expressed^18^ and essential for development^19^, BORIS expression is limited to the adult testis^18^, where it is involved in spermatogenesis. *BORIS*-null mice are subfertile with reduced testis size and delayed gamete production^20^,^21^. Various tumors and cancer cell lines also express BORIS, and so BORIS is considered to be a cancer-testis antigen^22^,^23^,^24^,^25^,^26^,^27^,^28^.

The existence of conditions under which both CTCF and BORIS, which have nearly identical DNA binding domains and thus bind the same DNA sequences^20^,^21^,^25^,^29^, are co-expressed led to speculation that they might compete for a finite number of binding sites^18^,^21^. However, mapping of CTCF and BORIS binding by ChIP-seq in human cancer and mouse germ cells revealed the existence of distinct classes of binding sites: 1xCTSes, containing a single motif and bound by CTCF alone (CTCF-only sites), and 2xCTSes, containing two or more clustered motifs and simultaneously bound by CTCF and BORIS (CTCF&BORIS sites) or BORIS alone (BORIS-only sites)^30^.

Despite the presence of multiple CTCF motifs within 2xCTSes, they display single peaks in ChIP-seq experiments, indicating that CTCF motifs generally lie too close together to be resolved by ChIP-seq^30^,^31^. Indeed, single motifs are usually reported for CTCF ChIP-seq peaks^32^, suggesting that 2xCTSes remain generally unexamined in genome-wide CTCF mapping studies. At CTCF&BORIS and BORIS-only sites, robust enrichment of RNA RNAPII, H3K4me3, and H3K27ac was observed, suggesting a function in active transcription. Furthermore, CTCF&BORIS and BORIS-only sites were associated with histone retention in mature spermatozoa, which has been proposed to be a consequence of active transcription in spermatids^33^ and is associated with genes expressed in early zygotic development^33^,^34^,^35^,^36^,^37^. These features contrast with 1xCTSes, which are not bound by BORIS, are generally located intergenically, and devoid of chromatin features associated with transcriptional activity^30^. Combined with the observation that BORIS-only sites do not overlap binding sites for cohesin, involved in long-range chromatin interactions and transcriptional insulation^38^,^39^, these data suggest that CTCF-only sites function in chromatin architecture, while BORIS-bound sites serve as transcriptional regulatory platforms. Indeed, prior to the genome-wide characterization of 2xCTSes, closely spaced CTCF motifs were described in several important regulatory regions including alternative human *BORIS* promoters^40^, the testis-specific *TSP50* promoter^41^, and the mouse KvDMR1 imprinting region^42^, suggesting that 2xCTSes are involved in important transcriptional regulatory mechanisms.

To better understand the potential functions of 2xCTSes, we undertook an integrative genomic analysis, incorporating ChIP-seq, RNA-seq, and MNase-seq data. We find that subsets of CTCF&BORIS and BORIS-only sites in mouse round spermatids are also occupied by a number of testis-specifc transcriptional regulators (TSTRs). These TSTR-occupied CTCF&BORIS (TSTR-CTCF&BORIS) and BORIS-only sites (TSTR-BORIS-only sites) are associated with genes highly expressed in testis and involved in male germ cell development. We also find that TSTR-associated BORIS-bound sites, but not their counterparts not bound by TSTRs, are associated with histone retention in mature sperm. Together, these observations suggest a functional link between CTCF and BORIS and other testis-specific TSTRs in the regulation of germ cell transcription. In support of this idea, we find that BORIS physically interacts with TATA-binding protein (TBP)-associated factor 7-like (TAF7L) in both cultured cells and human testis. Our analyses provide a refined view of BORIS-bound sites, identifying subsets of sites potentially involved in testis transcription and suggesting novel avenues for the exploration of functional connections between CTCF, BORIS, and several TSTRs in the regulation of male gametogenesis.

## Results

### TSTRs occupy a subset of CTCF&BORIS sites

**Figure 1:**
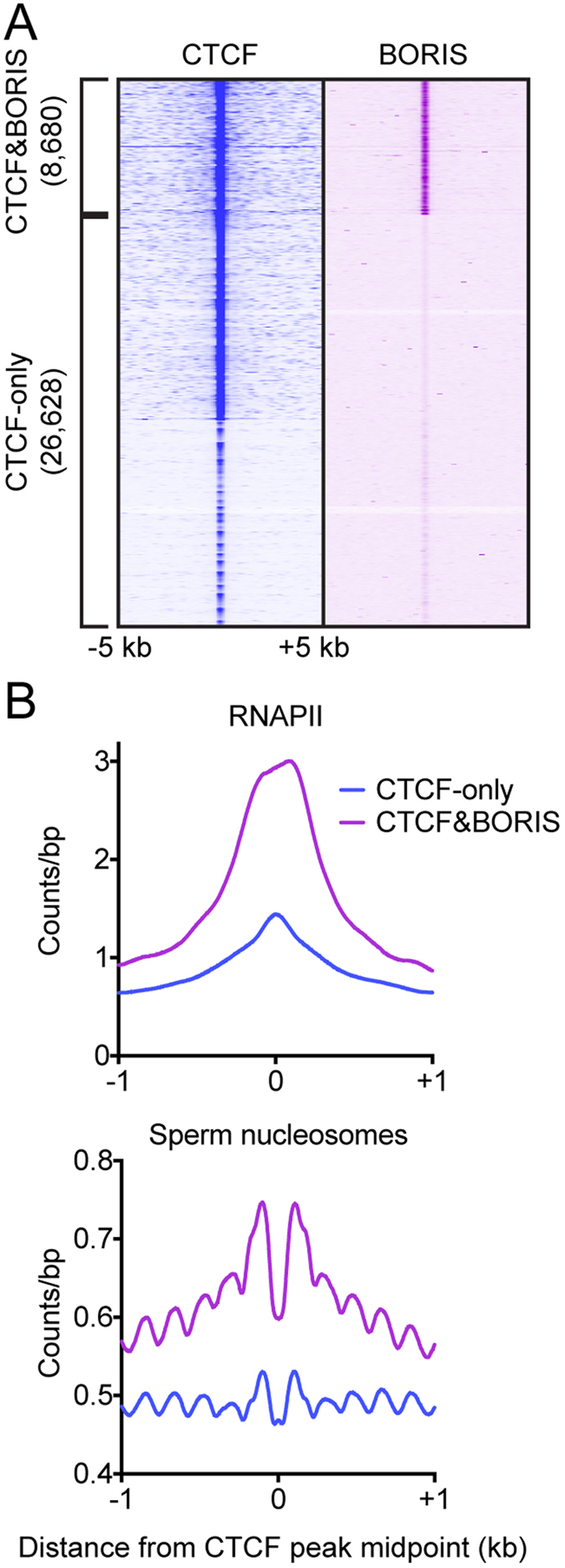
*De novo* identification and characterization of CTCF&BORIS sites in mouse round spermatids. (**A**) Heatmaps showing enrichment of CTCF and BORIS at 35,308 CTCF ChIP-seq peaks in mouse round spermatids. (**B**) Average plots of (top) RNAPII enrichment at CTCF-only and CTCF&BORIS sites in mouse testis and (bottom) nucleosome enrichment at CTCF-only and CTCF&BORIS sites in mouse spermatozoa.

To facilitate analysis of 2xCTSes, we first re-identified BORIS&CTCF sites in mouse round spermatids, making use of previously published CTCF and BORIS ChIP-seq data^30^. We identified 35,308 CTCF peaks, 8,680 (24.6%) of which were also occupied by BORIS (Fig. 1A), comparable to the ~8,500 round spermatid CTCF&BORIS sites identified in a previous study^30^. As we identified CTCF&BORIS sites using CTCF peaks as a reference, we first focused our analysis on a comparison of CTCF&BORIS to CTCF-only sites. Consistent with previously described properties of CTCF&BORIS sites^30^, we found that our CTCF&BORIS sites were, on average, more highly enriched than CTCF-only sites for both RNAPII binding in testis as well as nucleosomes in mature sperm (Fig. 1B). Consistent with previous work^43^, nucleosomes flanking CTCF-only and CTCF&BORIS sites were strongly phased.

Given that RNAPII is strongly enriched at CTCF&BORIS compared to CTCF-only sites^30^ (Fig. 1B), we hypothesized that CTCF&BORIS sites might also be bound by testis-specific transcriptional regulators (TSTRs) in addition to CTCF and BORIS. To test this idea, we analyzed ChIP-seq datasets for a number of TSTRs: A-MYB, a regulator of male meiosis^44^,^45^; BRD4, a bromodomain-containing factor that promotes elongation by RNAPII^46^; DMRT1, essential for testis development^47^; DMRT6, involved in spermatogonial differentiation^48^; TAF7L, a paralog of the general transcription factor TAF7 that is essential for spermiogenesis^49^,^50^; RFX2, essential for spermatid elongation^51^; and TRIM33, a poorly characterized transcriptional regulator (TR) containing a ubiquitin ligase domain, a bromodomain, and a plant homeodomain (PHD) finger responsible for silencing retrotransposons in the male germ line^52^.

**Figure 2:**
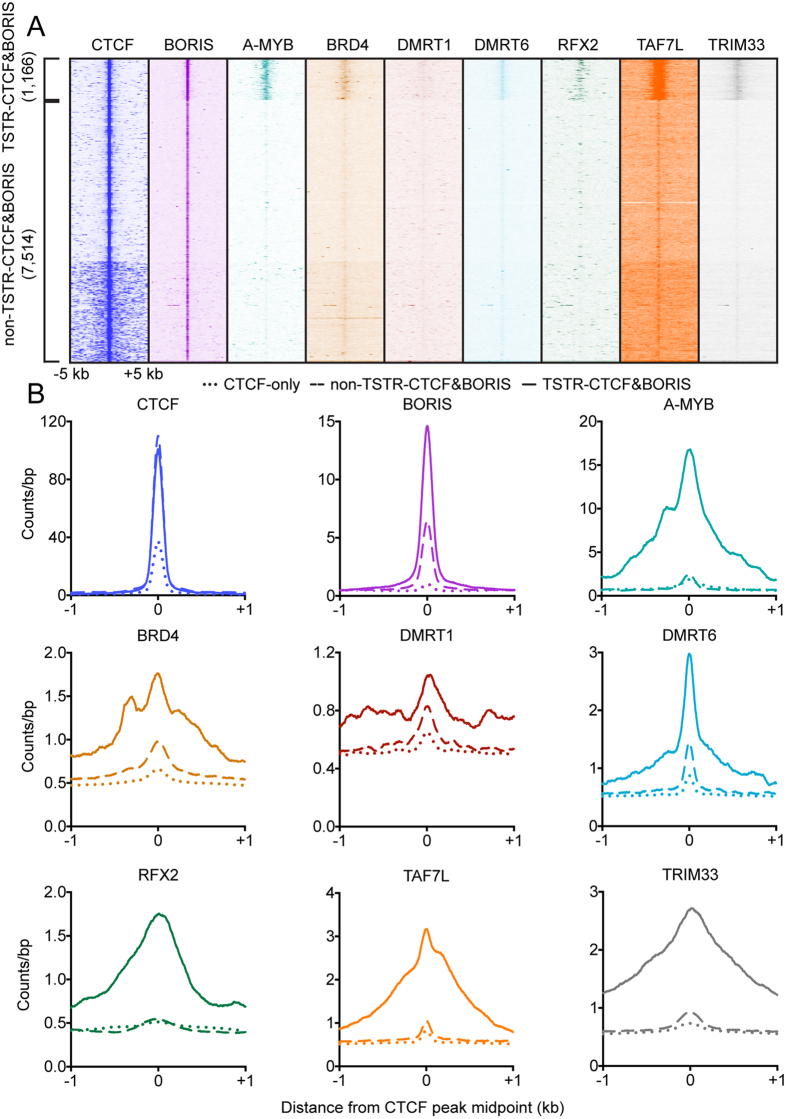
Testis-specific transcriptional regulators occupy a subset of CTCF&BORIS sites. (**A**) Heatmaps showing enrichment of CTCF, BORIS, A-MYB, BRD4, DMRT1, DMRT6, RFX2, TAF7L, and TRIM33 at 8,680 CTCF&BORIS sites. (**B**) Average plots of TR enrichment at CTCF-only, non-STTR-CTCF&BORIS, and TSTR-CTCF&BORIS sites.

We determined enrichment of each factor at CTCF&BORIS sites and then performed clustering, which revealed a striking partitioning of CTCF&BORIS sites into two classes. While the majority of CTCF&BORIS sites (7,514/8,680, 86.6%) displayed robust enrichment of CTCF and BORIS only, a substantial fraction (1,166/8,680, 13.4%) was also enriched for TSTRs (Fig. 2A, Supplementary Dataset 1). We refer this second class of sites as TSTR-associated CTCF&BORIS sites (TSTR-CTCF&BORIS sites). When datasets were scaled to identical read numbers and analyzed in aggregate, the most robustly enriched TSTRs were A-MYB, TAF7L, and DMRT6 (Fig. 2B). More modest enrichment was observed for BRD4, RFX2, and TRIM33, while little enrichment of DMRT1 was seen (Fig. 2B). Little to no enrichment of any TSTR was observed at CTCF-only sites (Fig. 2B). Of the identified TSTR-CTCF&BORIS sites, 693/1,166 (59.4%) were located between positions −1000 and +100 relative to the TSS, with the remainder located in untranslated regions, introns/exons, transcription termination sites, and intergenic regions. This observation suggests that TSTR-CTCF&BORIS sites are not simply due to a tendency for CTCF&BORIS sites to be located proximal to TSSs.

**Figure 3:**
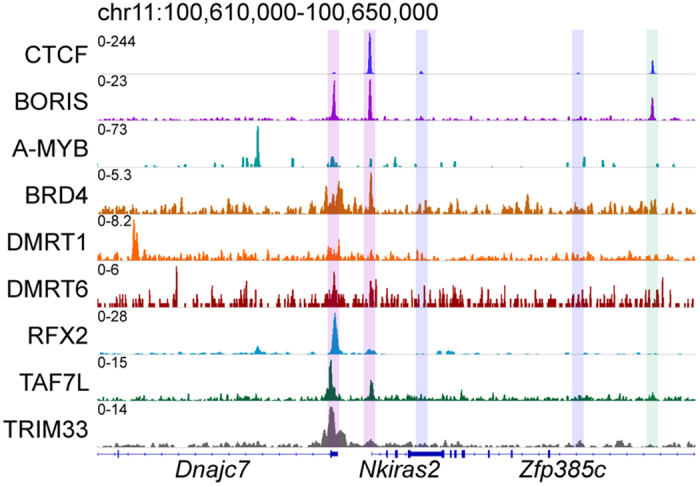
TSTR-CTCF&BORIS sites are occupied by various combinations of TSTRs. Genome browser visualization of a 40 kb segment of chromosome 11 showing two TSTR-CTCF&BORIS sites (purple), one non-TSTR-CTCF&BORIS site (green), and two CTCF-only sites (blue).

We next examined specific CTCF&BORIS sites through genome browser visualization. Analysis of a 40 kb segment of chromosome 11 revealed two TSTR-CTCF&BORIS, one non-TSTR-CTCF&BORIS, and two CTCF-only sites (Fig. 3). As expected from our average analysis (Fig. 2B), no TSTR enrichment was observed at the non-TSTR-CTCF&BORIS site or the CTCF-only sites (Fig. 3). We also noted different combinations of TSTRs at the TSTR-CTCF&BORIS sites. The site located at the promoter of *Dnajc7* displayed robust binding of BRD4, RFX2, TAF7L, and TRIM33 with lower occupancy of A-MYB, DMRT1, and DMRT6, while the site in promoter of the nearby *Nkiras2* gene was robustly bound by BRD4 and TAF7L, with little or no binding of A-MYB, DMRT1, DMRT6, RFX2, or TRIM33 (Fig. 3). Visualization of the 2xCTS-containing CTCF&BORIS and BORIS-only regions as single peaks regardless of TSTR occupancy highlights the previously described inability of ChIP-seq to resolve these closely spaced binding events in every analyzed mammalian cell type^30^.

### TSTR-CTCF&BORIS sites are associated with highly expressed male germ cell-specific genes

**Figure 4:**
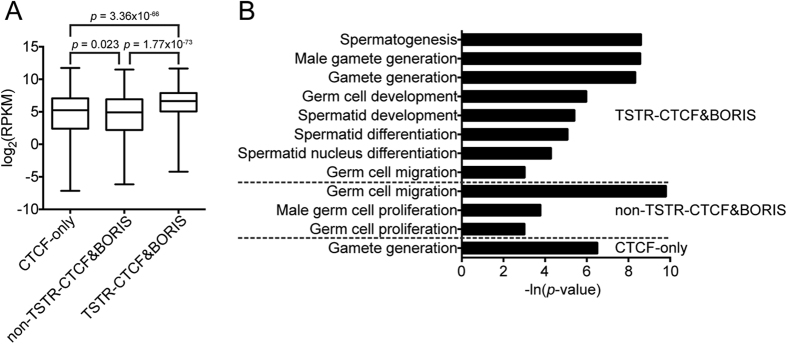
TSTR-CTCF&BORIS sites are associated with highly transcribed male germ cell-specific genes. (**A**) Boxplots of log_2_-transformed testis RNA-seq RPKM values for the closest gene to each CTCF-only, non-TSTR-CTCF&BORIS, and TSTR-CTCF&BORIS site. (**B**) Significant male or non-sex-specific germ cell-related GO terms for the nearest gene to each TSTR-CTCF&BORIS, non-TSTR-CTCF&BORIS, and CTCF-only site.

Given that TSTR-CTCF&BORIS sites are associated with TSTRs (Fig. 2), we anticipated that they might be associated with genes highly expressed in testis. We therefore analyzed the expression of genes associated with CTCF-only, non-TSTR-CTCF&BORIS, and TSTR-CTCF&BORIS sites using previously published testis RNA-seq data^50^. We found that genes associated with TSTR-CTCF&BORIS sites were expressed at a significantly higher level than those associated with either CTCF-only (*p* = 3.36 × 10^−66^ by *t*-test) or non-TSTR-CTCF&BORIS sites (*p* = 1.77 × 10^−73^ by *t*-test) (Fig. 4A). This is consistent with previous work suggesting association of BORIS&CTCF sites with active promoters and enhancers^30^. We next performed gene ontology (GO) analysis of genes associated with each category of site, focusing on germ cell-related annotations. This revealed significant association of eight distinct germ cell-related annotations with TSTR-CTCF&BORIS sites, compared with three for non-TSTR-CTCF&BORIS sites and one for CTCF-only sites (Fig. 4B). These observations support the idea that TSTR-CTCF&BORIS sites are involved in particular transcriptional programs specialized for both meiotic and post-meiotic developmental stages of spermatogenesis that support histone retention in mature sperm and thus facilitate transgenerational bookmarking of future promoters and enhancers on paternal DNA for post-fertilization zygotic genome activation (ZGA)^53^.

### TSTR-CTCF&BORIS sites are associated with histone retention in mature sperm

**Figure 5:**
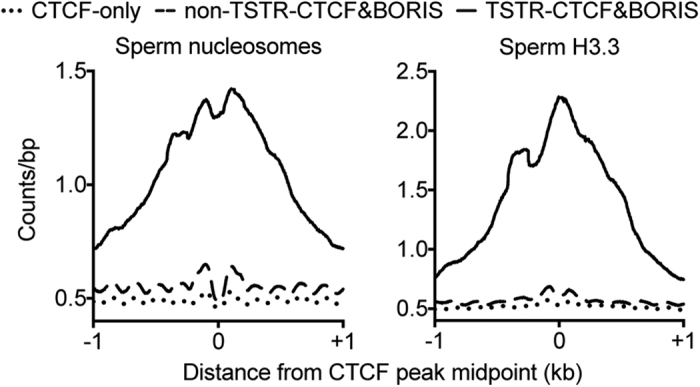
TSTR-CTCF&BORIS sites are associated with histone retention in mature sperm. Average plots of (left) nucleosome and (right) histone H3.3 enrichment in spermatozoa around CTCF-only, non-TSTR-CTCF&BORIS, and TSTR-CTCF&BORIS sites.

Previous work^30^ and our own analysis (Fig. 1B) indicate that CTCF&BORIS sites are, on average, enriched in regions that retain histones in mature spermatozoa after the large-scale replacement of histones for protamines has occurred. Given that previous work has suggested that robust transcriptional activity in spermatids is linked to histone retention^33^ and that TSTR-CTCF&BORIS sites are associated with highly expressed genes (Fig. 4A), we wondered if TSTR-CTCF&BORIS sites might also be preferentially associated with regions of histone retention in sperm. We therefore used MNase-seq data to assess the presence of nucleosomes at TSTR-CTCF&BORIS sites in spermatozoa. We found that TSTR-CTCF&BORIS, but not CTCF-only or non-TSTR-CTCF&BORIS, sites were associated with robust nucleosome retention in spermatozoa (Fig. 5). As nucleosome retention in sperm has been proposed to be a consequence of transcription-coupled deposition of histone H3.3 in spermatids^33^, we also assessed spermatozoal H3.3 enrichment. Consistent with the robust expression of TSTR-CTCF&BORIS site-associated genes, H3.3 was highly enriched at TSTR-CTCF&BORIS relative to CTCF-only and non-TSTR-CTCF&BORIS sites (Fig. 5).

### A subset of BORIS-only sites are occupied by TSTRs

**Figure 6:**
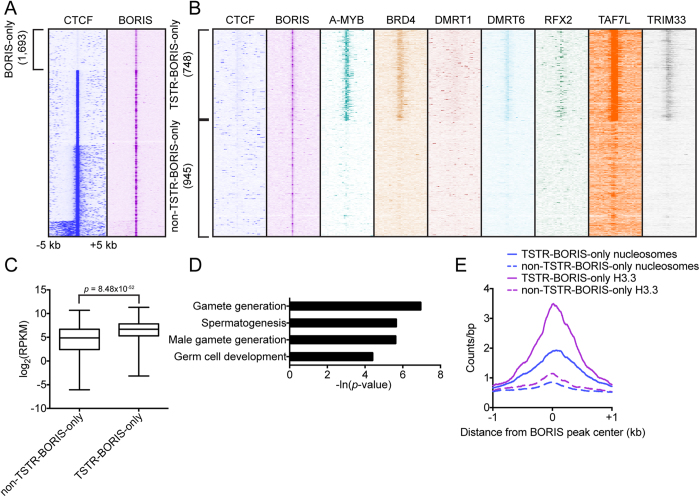
A subset of BORIS-only sites is associated with TSTRs. (**A**) Heatmaps showing enrichment of CTCF and BORIS at 8,644 BORIS ChIP-seq peaks in mouse round spermatids. (**B**) Heatmaps showing enrichment of CTCF, BORIS, A-MYB, BRD4, DMRT1, DMRT6, RFX2, TAF7L, and TRIM33 at 1,693 BORIS-only sites. (**C**) Boxplots of log_2_-transformed testis RNA-seq RPKM values for the closest gene to each non-TSTR-BORIS-only and TSTR-BORIS-only site. (**D**) Significant male and non-sex-specific germ cell-related biological process GO terms for the nearest gene to each TSTR-BORIS-only site (non-TSTR-BORIS-only sites displayed no enrichment of male or non-sex-specific germ cell-related GO terms). (**E**) Average plot of nucleosome and histone H3.3 enrichment in spermatozoa around TSTR-BORIS-only and non-TSTR-BORIS-only sites.

Thus far, we have shown that a fraction of CTCF&BORIS sites is also occupied by a number of TSTRs, and that these TSTR-CTCF&BORIS sites are linked to robust gene expression and retention of histones in sperm relative to non-TSTR-CTCF&BORIS and CTCF-only sites. However, 2xCTSes may also be occupied by BORIS alone (BORIS-only sites), and these BORIS-only sites display chromatin characteristics similar to those of CTCF&BORIS sites^30^. We therefore asked if some BORIS-only sites were also occupied by TSTRs. We called peaks on the BORIS ChIP-seq dataset and identified 1,693/8,644 (19.6%) sites not also bound by CTCF (Fig. 6A). We then quantified enrichment of A-MYB, BRD4, DMRT1, DMRT6, RFX2, TAF7L, and TRIM33 around BORIS-only sites and performed clustering. We found that 748/1,693 (44.2%) of BORIS-only peaks were also occupied by TSTRs (Fig. 6B). We note that the proportion of such TSTR-BORIS-only sites in BORIS-only sites is much higher than that of TSTR-CTCF&BORIS in CTCF&BORIS sites (44.2% vs. 13.4%). Of the identified TSTR-BORIS-only sites, 455/748 (60.8%) were located at the promoter/TSS regions, suggesting that, similar to TSTR-CTCF&BORIS sites, TSTR-BORIS-only sites are not simply the result of a locational bias. Genes associated with TSTR-BORIS-only sites were more highly expressed than those associated with non-TSTR-BORIS-only sites (*p* = 8.48 × 10^−52^ by *t*-test) (Fig. 6C), and genes associated with TSTR-BORIS-only sites were strongly linked to male germ cell biological process annotations (Fig. 6D). Like TSTR-CTCF&BORIS sites, TSTR-BORIS-only sites showed strong retention of nucleosomes bearing histone H3.3 in mature spermatozoa (Fig. 6E).

### BORIS and TAF7L physically interact *in vitro* and *in vivo*

**Figure 7:**
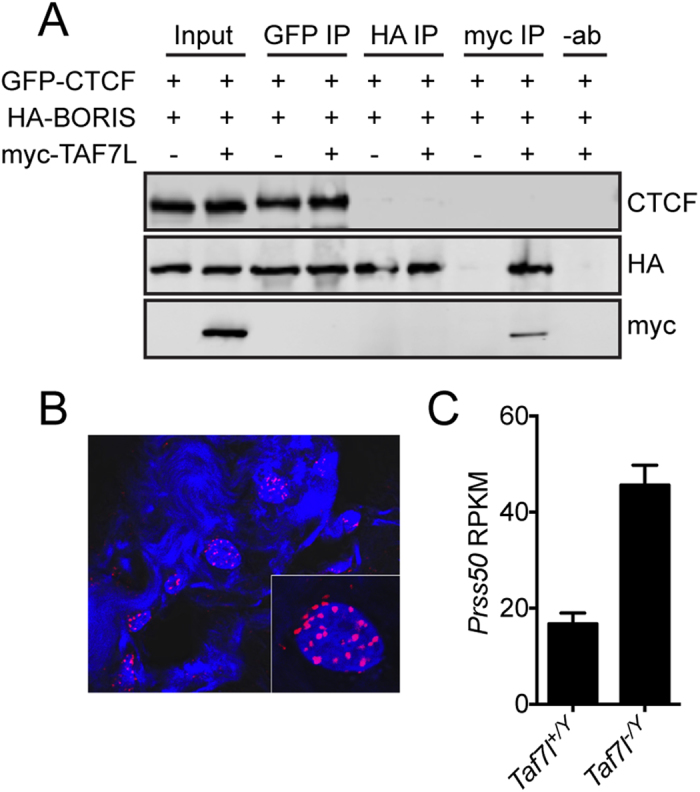
BORIS and TAF7L physically interact *in vitro* and *in vivo.* (**A**) Western blots detecting GFP-CTCF, HA-BORIS, and myc-TAF7L following co-IP with the indicated antibodies in HEK293T cells. **(B**) *In situ* proximity ligation assay (ISPLA) for BORIS and TAF7L in human testis. (**C**) Expression of *Prss50* in *Taf7l*^+/*Y*^ and *Taf7l*^−/*Y*^ mouse testes. Error bars represent mean RPKM +95% CI.

The genomic colocalization of BORIS with several TSTRs with or without concomitant CTCF binding led us to investigate potential physical interactions between these proteins. We focused on TAF7L, a paralog of the TFIID component TAF7 that is essential for spermiogenesis^49^, as it is localized to the nucleus in round spermatids^54^, from which the CTCF and BORIS ChIP-seq data were generated^30^. We expressed HA-tagged BORIS, GFP-tagged CTCF, and myc-tagged TAF7L in HEK293T cells and performed co-IPs. BORIS but not CTCF was efficiently co-IPed with TAF7L, though we were unable to detect reciprocal co-IP of TAF7L with BORIS despite repeated attempts (Fig. 7A). To confirm the observation of BORIS/TAF7L physical interaction in cultured cells *in vivo*, we performed an *in situ* proximity ligation assay (ISPLA) on frozen human testis. Numerous fluorescent foci, indicative of spatial proximity, were observed (Fig. 7B), further suggesting a physical interaction between BORIS and TAF7L, though we were unable to determine the cell type in which these interactions occurred. To investigate a functional connection between TAF7L and BORIS in gene regulation, we first examined the effect of TAF7L loss on the expression of *Prss50*, a well-characterized BORIS target gene^29^. Interestingly, *Taf7l*^*−/Y*^ testes displayed a ~2.7-fold increase in *Prss50* compared to wild type (Fig. 7C), perhaps suggesting that TAF7L acts to restrain transcriptional activation by BORIS.

**Figure 8:**
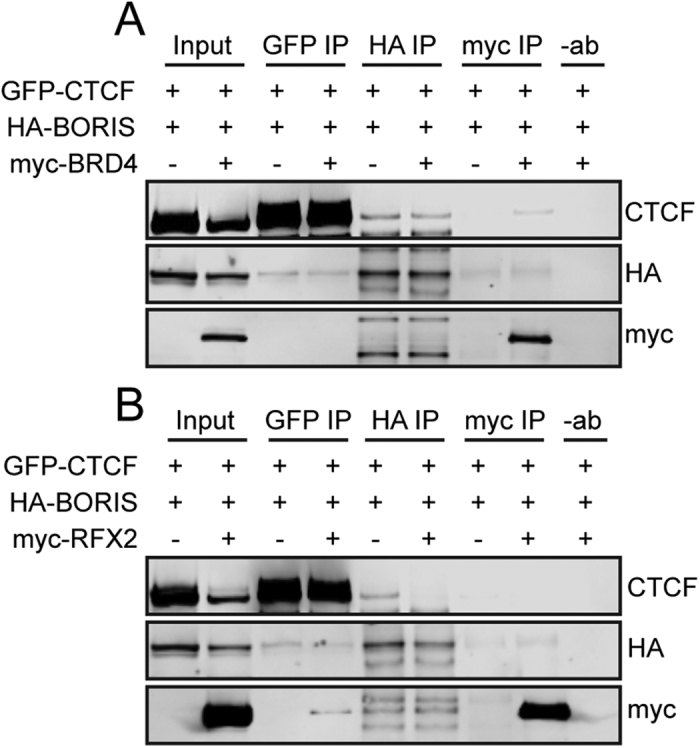
Analysis of physical interactions of CTCF and BORIS with BRD4 and RFX2. (**A**) Western blots detecting GFP-CTCF, HA-BORIS, and myc-BRD4 following co-IP with the indicated antibodies in HEK293T cells. (**B**) Same as (**A**), but with myc-RFX2.

We also examined potential physical interactions between CTCF and BORIS and two additional TSTRs, BRD4 and RFX2. We detected weak co-IP of CTCF with BRD4, though the reciprocal result was not observed (Fig. 8A). Similarly, we detected a small amount of RFX2 in a CTCF IP, though no CTCF was observed in an RFX2 IP (Fig. 8B). We note that the relative weakness or absence of these interactions does not necessarily preclude their taking place in germ cells, as cofactors and/or post-translational modifications necessary for their interaction may not be present in HEK293T cells.

## Discussion

CTCF and its paralog BORIS occupy thousands of sites, both individually and in combination, throughout the genome in mouse male germ cells. Sites bound by BORIS, both with and without CTCF, are not well understood. Here, we integrated ChIP-seq, RNA-seq, and MNase-seq data into a genomic characterization of CTCF&BORIS and BORIS-only sites. We found that substantial fractions of CTCF&BORIS and BORIS-only sites are also occupied by TRs with known roles in male gametogenesis and associated with genes highly expressed in testis as well as sites of histone retention in spermatozoa.

Our results place BORIS and CTCF in the context of a larger germ cell-specific transcriptional network, suggesting that they cooperate with a number of testis-specific TSTRs potentially capable of physically interacting with CTCF and BORIS through protein-protein interactions with either the common central 11-ZF region or their unique C- and N-termini. At first approximation, it may appear that BORIS is a relatively minor component of this transcriptional network, as only a handful of genes have been identified as dysregulated in *BORIS*-null testes^20^,^21^, with only two such genes (*Prss50, Gal3st1*) reported to be affected in both studies. However, it is known that CTCF can partially compensate for BORIS loss in mouse ES cells^21^ and that BORIS-only 2xCTSes are occupied by CTCF homodimers in BORIS-negative cells^30^. Additionally, a recent study showed that while conditional knockout of *Ctcf* in male germ cells decreased the expression of 2,549 genes, only a small fraction of these genes displayed CTCF binding to their promoter or a nearby putative enhancer^55^, suggesting that CTCF alone directly regulates the expression of relatively few genes. We note the possibility that homodimerization of CTCF or CTCF/BORIS heterodimerization might be responsible for the formation of some TSTR-bound regions, as occupancy of TSTRs at CTCF-only sites, occupied by a CTCF monomer, is not observed. In this model, in *BORIS*-null testes, CTCF homodimers occupy 2xCTSes normally bound by CTCF/BORIS heterodimers or BORIS homodimers, allowing the formation of TSTR-bound regions and limiting the transcriptional effects of BORIS loss. However, because not all 2xCTSes are TSTR-occupied, there must be additional factors beyond the dimerization of CTCF and/or BORIS that promote TSTR binding at these sites. Moreover, the effect of conditional *Ctcf* knockout on male mouse fertility is more severe than that of *BORIS* deletion^20^,^21^,^55^. Future studies comparing the transcriptional effects of CTCF and BORIS loss alone and in combination will be informative in understanding potential redundancy and specialization between these factors. One interesting direction to pursue in this regard may be *in vivo* analysis of the regulation of the TFIIAα/β-like factor (ALF) gene, encoding a germ cell-specific component of TFIIA^56^, the promoter of which is differentially regulated by CTCF and BORIS in cultured cells^57^. It may also be of interest to analyze chromatin interactions in germ cells expressing various levels of CTCF and BORIS. Fusion of the CTCF central 11-ZF domain to a different N-terminus, thereby creating a CTCF decoy with altered protein-protein interaction capabilities, causes disruptions in chromatin looping and imprinting at the human *IGF2* locus^58^. Given that the central 11-ZF regions of CTCF and BORIS are highly conserved but their N- and C-termini very divergent^18^, BORIS could potentially serve as a decoy for CTCF, altering chromatin interactions. Indeed, BORIS-only regions do not overlap with peaks for cohesin, thought to be generally required for long-range chromatin interactions^39^, in human cancer cells^30^, suggesting that expression of BORIS can alter the chromatin interaction landscape of a cell. Beyond analysis of redundancy and specialization between CTCF and BORIS, comparison of the transcriptional profiles of germ cells with single and combined BORIS and CTCF deficiency to those harboring mutations in other TSTRs associated with CTCF&BORIS and BORIS-only sites, such as TAF7L, shown here to physically interact with BORIS, may be a productive direction for understanding the transcriptional roles of CTCF and BORIS in male germ cells.

We note that while the CTCF and BORIS ChIP-seq data were generated using round spermatids, the TSTR ChIP-seq datasets, with the exception of BRD4, were generated using whole testis. It is therefore possible that our analyses were confounded by the presence of additional cell types. However, there is evidence that several of the analyzed factors function in round spermatids. TAF7L physically interacts with BORIS but not CTCF, suggesting a germ cell-exclusive role for this interaction, and is localized to the nucleus of round spermatids^54^, providing further evidence that genomic colocalization with CTCF and BORIS does occur in these cells. There is also evidence that the other TSTRs analyzed here function in round spermatids. A-MYB can be detected in early round spermatids^59^, and RFX2, itself present in round spermatids^60^ and required for their elongation^51^, has been proposed to be an amplifier of A-MYB regulation^59^. TRIM33 is also expressed in round spermatids^61^. Additionally, DMRT1, which was not found at TSTR-occupied regions, is not detected in spermatids^62^. Its absence from TSTR-occupied sites may therefore indicate that these regions are specific to spermatids. However, ChIP-seq mapping of additional TSTRs in round spermatids is necessary to conclusively address this.

Our data also bear on the proposed role of BORIS-bound regions as markers for histone retention in mature spermatozoa^30^. We found that, of the identified CTCF&BORIS and BORIS-only sites, only those regions that were associated with TSTRs were strongly linked to protamine-refractory, histone-retaining regions in mature sperm. It has been suggested that ongoing transcription and concomitant deposition of the H3.3 histone variant in spermatids is a trigger for histone retention during the histone-to-protamine transition in the haploid phase of spermatogenesis^33^. Indeed, we found that TSTR-CTCF&BORIS and TSTR-BORIS-only sites were associated both with highly expressed genes in testis and H3.3 occupancy in sperm, suggesting that retention of histones at BORIS binding sites in sperm is dependent on cooperation with TSTRs to generate high levels of transcription. TSTR-marked BORIS-bound sites in spermatids could therefore contribute to histone retention in sperm by promoting high levels of transcription in spermatids and also organizing sperm chromatin. However, it is unclear if BORIS is expressed in spermatozoa, so these regions might be bound by CTCF homodimers. CTCF protein is present in sperm^63^, nuclease-sensitive regions in human spermatozoal chromatin are enriched in CTCF binding motifs^35^, and there is potentially widespread occupancy of CTCF in mouse spermatozoa^64^,^65^, suggesting a role for CTCF in organizing sperm chromatin. Indeed, CTCF-deficient spermatozoa display defects in chromatin condensation^55^. Regardless, ChIP-seq profiling of CTCF and BORIS in spermatozoa will be necessary to determine the contribution of TSTR-associated BORIS binding sites to sperm chromatin organization.

Our results provide a refined view of the nature of BORIS-bound sites in the mouse male germ cell genome, indicating that subsets of these sites are potentially involved in promoting testis transcription through the synergistic action of CTCF and BORIS with a number of TSTRs. We find that the presence of a CTCF&BORIS or BORIS-only site is in and of itself insufficient for histone retention in spermatozoa, but suggest that synergy between CTCF and/or BORIS and additional TSTRs provides a high level of transcription that leads to histone H3.3 deposition and subsequent retention of histones after the histone-to-protamine transition. This suggests that TSTR-occupied BORIS-bound regions function in the maintenance of transgenerational bookmarks at promoters and enhancers to be activated during ZGA following fertilization.

## Methods

### Datasets

All datasets were obtained from the SRA. ChIP-seq: A-MYB, whole testis^44^ (SRX244353); BORIS, round spermatids^30^ (SRX1091833); CTCF, round spermatids^30^ (SRX1091832); BRD4, round spermatids^46^ (SRX719833); DMRT1, whole testis^47^ (SRX838549); DMRT6, whole testis^48^ (SRX681861); (SRX112976); Histone H3.3, mature spermatozoa^33^ (SRX207534); input, round spermatids^30^ (SRX1091834); RFX2, whole testis^51^ (SRX1011026); RNAPII, whole testis^50^ (SRX349391); TAF7L, whole testis^50^ (SRX349390); TRIM33, whole testis^52^ (SRX1019997); MNase-seq: mature spermatozoa^33^ (SRX207533); RNA-seq: whole testis^50^ (SRX349392).

### Data analysis

#### Alignment

ChIP-seq and MNase-seq data were aligned to the mm10 mouse genome build using Bowtie2^66^ with default parameters. RNA-seq data were aligned to the mm10 mouse genome build with STAR^67^ version 2.4.1 d using the corresponding refGene GTF file from the UCSC Table Browser.

#### Peak calling

Tag directories were created from CTCF, BORIS, and input aligned SAM files with HOMER^68^ (http://homer.salk.edu). Peaks were called with HOMER findPeaks using –style factor and round spermatid input as control. CTCF and BORIS peaks are listed in Additional File 1, Tabs 1 and 2.

#### Cluster analysis and heatmaps

Aligned SAM files were converted to BAM format with SAMTools^69^ and input into SeqMiner^70^ for clustering. We used linear *k*-means clustering with *k* = 3 of the CTCF and BORIS datasets around CTCF peaks to identify CTCF-only and CTCF&BORIS sites (Supplementary Dataset 1, Tabs 3 and 4). We then used ranked *k*-means clustering with *k* = 4 of all datasets around CTCF&BORIS sites to identify CTCF&BORIS sites bound or not bound by additional transcriptional regulators (TSTR-CTCF&BORIS and non-TSTR- CTCF&BORIS sites, Supplementary Dataset 1, Tabs 5 and 6). We used linear *k*-means clustering with *k* = 2 of the CTCF and BORIS datasets around BORIS peaks to identify BORIS-only sites (Supplementary Dataset 1, Tab 7). We then used ranked *k*-means clustering with *k* = 2 of all datasets around BORIS-only sites to identify BORIS-only sites bound or not bound by additional transcriptional regulators (TSTR-BORIS-only and non-TSTR-BORIS-only, Supplementary Dataset 1, Tabs 8 and 9). All heatmaps were generated with SeqMiner using a contrast value of 50.

#### Average plots

The annotatePeaks.pl feature of HOMER was used to generate average plots around CTCF-only and CTCF&BORIS sites with 1 bp resolution. Default normalization (scaling of tag directories to 10 million reads) was used.

#### Genome browser visualization

HOMER makeUCSCfile was used to generate bedgraphs normalized to 10 million reads. Bedgraphs were converted to bigwig format and visualized in IGV^71^.

#### Transcript quantification

HOMER analyzeRepeats.pl was used to quantify RNA-seq reads in exons, allowing a maximum of 3 tags per position (–pc 3) and using gene expression matrix normalization (–normMatrix 1e7).

#### Gene ontology analysis

GO analysis was performed with HOMER annotatePeaks.pl. The –ln(*p*-value) of each biological process annotation related to male germ cell development was reported.

### Co-immunoprecipitation (co-IP)

HEK293T cells were grown in DMEM + 10% FBS and transfected with pCI-Neo-GFP-CTCF and pCI-Neo-HA-BORIS along with pCI-Neo-myc-TAF7L, pReceiver-myc-BRD4 (Genecopoeia), or pReceiver-myc-RFX2 (Genecopoeia). For co-IP analysis, 1.5 mg of total protein was incubated with anti-Myc antibody (Sigma), anti-HA antibody (Roche), or anti-GFP antibody (Abcam ab290) overnight at 4 °C with gentle agitation, followed by incubation with 50 μl of a 1:1 mixture of Protein A and Protein G Dynabeads (Life Technologies) for 1 h at room temperature. The immunoprecipitates were collected using a magnetic rack and washed and analyzed by SDS-PAGE and western blotting.

### *In situ* proximity ligation assay (ISPLA)

Frozen human testis slides were fixed in 4% paraformaldehyde for 10 min at 37 °C. Slides were then permeabilized for 2 h with 0.2% Triton X-100 in PBS then blocked in 3% bovine serum albumin (Sigma) in a humid chamber for 1 h at 37 °C and incubated overnight at 4 °C with mouse and rabbit antibodies: custom mouse monoclonal anti-BORIS and rabbit anti-TAF7L (Santa-Cruz, S-24) in blocking solution. After washing, the slides were incubated with Duolink PLA Rabbit MINUS and PLA Mouse PLUS probes. Ligation and detection were performed using the Duolink reagents kit (Olink Bioscience, Sigma) according to the manufacturer’s protocol. Fluorescence was detected using a Zeiss Plan Apochromat microscope with a 63x/oil objective. Images were acquired with an Axiocam MRm camera and the Zeiss LSM Image Browser. Presence or absence of foci in specific testis cell types was not assessed.

## Additional Information

**How to cite this article**: Rivero-Hinojosa, S. *et al*. Testis-specific transcriptional regulators selectively occupy BORIS-bound CTCF target regions in mouse male germ cells. *Sci. Rep.* **7**, 41279; doi: 10.1038/srep41279 (2017).

**Publisher's note:** Springer Nature remains neutral with regard to jurisdictional claims in published maps and institutional affiliations.
