# Corrigendum: Testis-specific transcriptional regulators selectively occupy BORIS-bound CTCF target regions in mouse male germ cells

**DOI:** 10.1038/srep46891

**Published:** 2017-08-04

**Authors:** Samuel Rivero-Hinojosa, Sungyun Kang, Victor V. Lobanenkov, Gabriel E. Zentner

Scientific Reports 7: Article number: 41279; 10.1038/srep41279 published online: 02 01 2017; updated: 08 04 2017.

In the original version of this Article, Supplementary Dataset 1 was omitted. This has been corrected in the HTML version of the Article; the PDF version was correct at the time of publication.

